# Influence of Stress Intensity Factor on Rail Fatigue Crack Propagation by Finite Element Method

**DOI:** 10.3390/ma14195720

**Published:** 2021-09-30

**Authors:** Ruipeng Gao, Mengmeng Liu, Bing Wang, Yiran Wang, Wei Shao

**Affiliations:** 1 School of Mechanical and Precision Instrument Engineering, Xi’an University of Technology, Xi’an 710048, China; vessjessy@xaut.edu.cn (R.G.); 18791987859@163.com (M.L.); lm18049060527@163.com (B.W.); 2 State Key Laboratory for Mechanical Behavior of Materials, School of Materials Science and Engineering, Xi’an Jiaotong University, Xi’an 710049, China

**Keywords:** wheel-rail rolling contact fatigue, ANSYS, three-dimensional, rail crack length, crack propagation angle

## Abstract

Wheel rail rolling contact fatigue is a very common form of damage, which can lead to uneven rail treads, railhead nuclear damage, etc. Therefore, ANSYS software was used to establish a three-dimensional wheel–rail contact model and analyze the effects of several main characteristics, such as the rail crack length and crack propagation angle, on the fatigue crack intensity factor during crack propagation. The main findings were as follows: (1) With the rail crack length increasing, the position where the crack propagated by mode I moved from the inner edge of the wheel–rail contact spot to the outer edge. When the crack propagated to 0.3–0.5 mm, it propagated to the rail surface, causing the rail material to peel or fall off and other damage. (2) When the crack propagation angle was less than 30°, the cracks were mainly mode II cracks. When the angle was between 30 and 70°, the cracks were mode I–II cracks. When the angle was more than 70°, the cracks were mainly mode I cracks. When the crack propagation angle was 60°, the equivalent stress intensity factor reached the maximum, and the rail cracks propagated the fastest.

## 1. Introduction

With the accelerated development of both high speed and heavy loads railway transportation, wheel rail rolling contact fatigue (RCF) damage has become increasingly serious [1]. Under heavy loads, rails develop fatigue microcracks, which propagate to macrocracks [2]. When fatigue macrocracks propagate to a certain extent, they will eventually lead to sudden rail fractures, casualties, and other catastrophic consequences [3]. Therefore, the study of rail fatigue crack propagation characteristics is helpful for analyzing the crack failure mechanism to provide a theoretical basis for improving railway transportation safety.

Rail materials are fractured by the formation of displacement discontinuities therein. Depending on the stress conditions, rail cracks can be categorized as opening mode (mode I), sliding mode (modeII), or tearing mode (mode III) [4]. In practical engineering applications, the mode of force exerted on a rail is very complex when a wheel and rail are in contact, that is, the force cannot be characterized by only a single mode, rather, it may be characterized by a mixed-mode crack of two or more modes. Therefore, the resulting cracks are mixed-mode cracks.

G.R. Irwin [5] first proposed the concept of the stress intensity factor to quantify the magnitude of the stress field at crack tips and predict the crack propagation trend. It determines whether the crack will destabilize and propagate and the direction of propagation and, on this basis, it establishes fracture toughness-related theoretical systems. The theory which G.R. Irwin proposed connects crack fractures with stress intensity factor at crack tips so that fractures can be quantified.

Researchers have conducted many studies on rail fatigue crack propagation. For example, Wang et al. [6] proposed an analytic solution for the stress intensity factor of symmetric coalescence cracks, and then the effect of the crack propagation direction and the geometry characteristics on the stress intensity factor was discussed. The results show that the stress intensity factor decreased and then remained at an asymptotic value with the increase in the intersection angle. Simultaneously, the magnitudes of the stress intensity factors for the mode−I and mode−II cracks decreased in the order of the diagonal crack, the central crack, and the edge crack. To understand the phenomena of propagation of macro−cracks in the core and delamination at the face−to−core interface, Funari et al. [7] studied in depth the research topics of “On the elastic and mixed−mode fracture properties of PVC foam”. Zan et al. [8] used a two−dimensional rail finite element model to simulate the change in the plastic zone at the tip of rail surface cracks for various numbers of load cycles. The simulation results showed that when the friction between the wheel and the rail was low (e.g., μ=0.1, where μ is dynamic friction factor), the size of the plastic zone at the crack tip met the “small range” yield condition of linear elastic fracture mechanics. When the friction between the wheel and the rail was high (e.g., μ=0.2), the cyclic plasticity at the crack tip increased significantly, and the cyclic plastic zone at the crack tip of a large angle crack (e.g., θ=30°, where θ is the angle between the rail crack and the train−wheel running direction) was significantly larger than that at the crack tip of a small angle crack (e.g., θ=15°). Elsayed et al. [9] used an elastic−plastic finite−element framework to investigate the response of rail material, and cracks initiated at the rail surface knowing that the simulated friction coefficient between wheel and rail is 0.35 and the applied wheel load is 110 kN. Additionally, 15 mm depth is enough to study the nonlinear characteristics of rail materials. Ma et al. [10] developed a two−dimensional method of predicting the initiation of railhead fatigue cracks by analyzing reasonable model parameters and verifying the method accuracy by comparing the obtained results with those obtained using classical continuum mechanics and those previously published in the literature. They analyzed and discussed the effects of wheel−rail rolling and sliding, friction coefficients, and wheel loads on fatigue crack initiation. 

In summary, although the effects of crack length and angle on rail contact fatigue cracks cannot be ignored, the two-dimensional model adopted by most scholars may ignore some three-dimensional factors [11]. With the rapid development of computer technology [12] and finite element simulations [13], three-dimensional crack propagation must be studied under complex loads [14]; few people have researched this field at present. Therefore, elucidating the influence of the number of cyclic loads on the propagation of microcracks on rail treads during rail fatigue crack growth, preliminary research combined ANSYS finite element analysis as well as Siemens NX three-dimensional drawing software to establish a wheel–rail three-dimensional contact-fatigue model and found that under horizontal and vertical cyclic loads, the rail crack length increased with increasing number of loads, the effect of the cyclic loads was logarithmic, and the fitting accuracy reached 0.992, which is consistent with the Paris curve [15]. Therefore, the use of ANSYS software to establish a three-dimensional wheel–rail contact model can analyze the effects of several main characteristics, such as the rail crack length and crack propagation angle, on the fatigue crack intensity factor during crack propagation.

In this paper, the wheel–rail three-dimensional contact-fatigue model was used to analyze the rail fatigue contact problem under different factors of crack length and crack angle. Firstly, a three-dimensional wheel–rail contact-fatigue model was established by ANSYS finite element combined with the software Siemens NX. Secondly, the influence of different factors was studied such as crack length and crack angle on rail contact fatigue cracks. Finally, the law of crack growth under different factors is obtained. The research in this paper obtained the results of the influence of rail crack characteristics on the rail fatigue crack intensity factor, which played an important theoretical support role for the subsequent wheel rail protection.

## 2. Model Establishment

The similarity between the stress intensity factor directly calculated by finite element ANSYS and the value obtained by fracture mechanics can reach 99.9 %. Therefore, in the linear elastic range of rail, using ANSYS to solve the stress intensity factor of rail crack tip has high accuracy [16]. When calculating the stress intensity factor, the closer the selected point is to the crack tip region, the more accurate the calculation results are, so the feasibility of using finite element analysis to study the crack propagation problem is illustrated.

In this paper, we use ANSYS to establish a plane model, then stretch it into a body, apply boundary constraints, and finally calculate the stress intensity factor.

According to the Chinese standard requirements, GB 2585-2007 for wheels and rails, the wheel material and track materials used in this model are SMA490B and U71Mn, respectively. The specific wheel and rail properties [15] are listed in Table 1.

According to China’s iron rail standards GB 2585-2007, the type of rail is 60 kg/m, and the rail’s height is 176 mm. In this paper, the software ANSYS and Siemens NX were used to establish the rail contact model, and the type of rail was selected as 60 kg/m, as per the requirements of the Chinese standard. Firstly, the software Siemens NX was used to establish the 3D rail model, which was subsequently imported into ANSYS to configure the rail contact. Generally, the load applied to the rail can be divided into three types: (1) vertical load, that is, the load perpendicular to the rail, this part of the load is generated by the weight of the train and the cargo; (2) friction, the force between the wheels and the rails due to the process of starting and braking the vehicle; (3) The traction torque of the train, that is, the torque generated by the traction of the train during the running process. To reduce the amount of calculation, the model is simplified in this paper. The specific part is ignoring factors such as roadbed, crosstie, and other factors. The steel rails mainly bear three kinds of forces from the axial force of the train, the friction between the wheels and the rail, and the traction force from the train. In the three-dimensional model, the train wheels are concentrated at the center node of the wheel, and then the vertical load is applied to the node position, friction is added to the center point of the contact spot, and traction torque is applied to the center node of the wheel.

Among them, the axial pressure is the weight of the train and the cargo, the friction force is the friction force generated by the wheel and rail during the starting and braking process, and the torque is the traction force experienced by the train during the driving process. The boundary conditions of the wheel–rail three-dimensional model are: the two sides and bottom of the rail are fully constrained.

**Figure 1:**
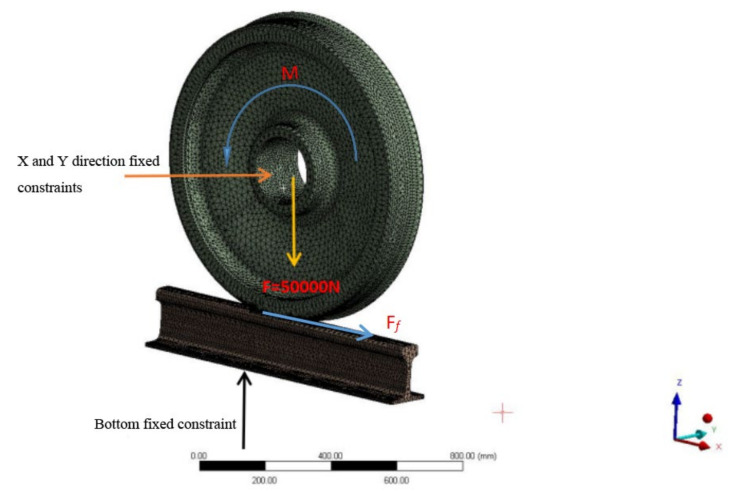
Three-dimensional rail contact model.

The results of the 3D rail contact model are shown in Figure 1 [15], and a force of F=−50,000 N is added to the hub bore along the *z*−direction, a fixed restraint is added to the track in the positive direction of the *z*-direction, a fixed restraint is added to the inside of the hub along with the *x* and *y*-directions as indicated by the arrow, a torque is added to the wheel, the lateral displacement is 0 mm, the shaking head angle is 0 radians, and the traction force is set to 1.8 × 105 N.mm.

**Figure 2:**
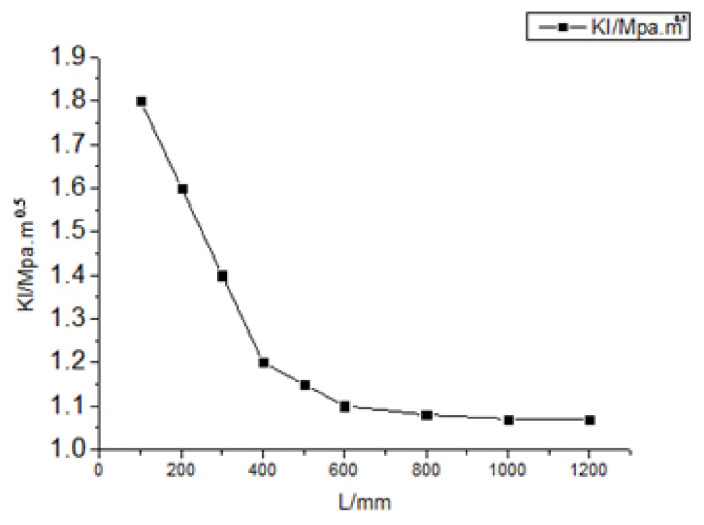
The variation of stress intensity factor $K_{I}$ with the length of the rail model.

To reduce the number of calculations and save calculation time, the appropriate rail model length should be selected, which can be obtained by conducting a series of test experiments as follows. Under an axle load of 5 t, the distance between the center position of a contact spot and a crack was maintained at 5.6 mm, and $K_{I}$ (where $K_{I}$ is the stress intensity factor for a sliding crack at the rail crack) was calculated for L (where L is the length of the rail model) in the range 100−1200 mm. The results are shown in Figure 2 [15].

According to Figure 2, $K_{I}$ gradually decreases with increasing L and stabilizes when L=600 mm. Therefore, L=600 mm was selected to reduce the number of calculations and calculation time.

The SOLID 186 element, which has 20 nodes, was selected to model the rail material, and each node has three degrees of freedom in the *x*-, *y*-, and *z*-directions.

**Figure 3:**
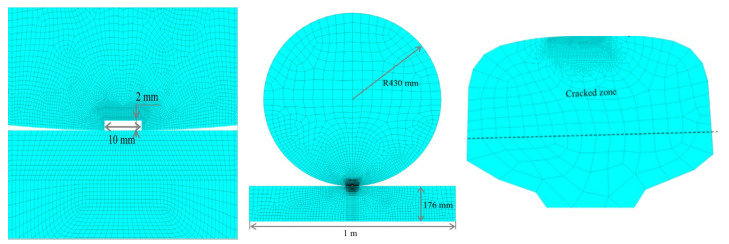
Local mesh diagram of the steel-rail model.

**Figure 4:**
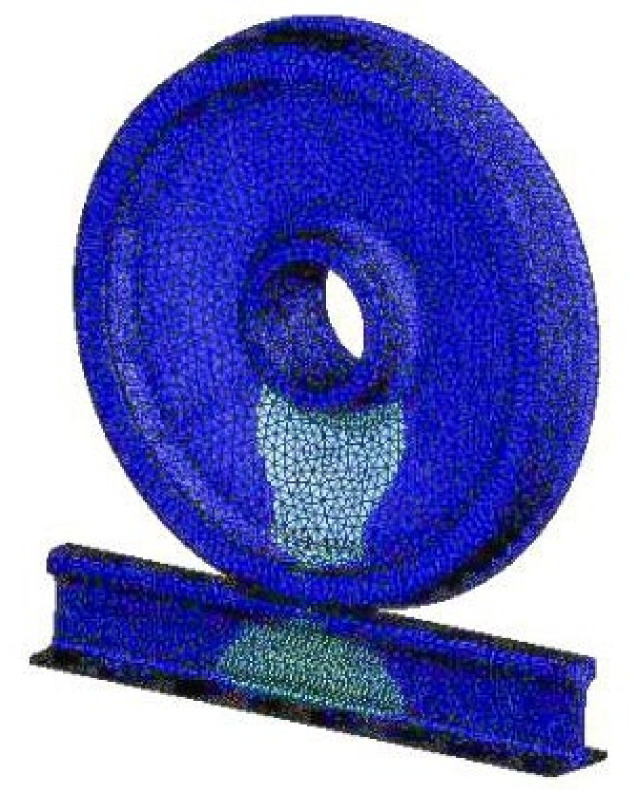
ANSYS rail model.

Because the main analysis in this study was the propagation of fatigue cracks on rail treads, the wheel model mesh was relatively sparse. The rail cracks were finely divided into meshes, and the local meshes were subdivided in the crack tip area. When the rail contact model has meshed, the wheel was divided by a triangular mesh. Because the rail was the focus of the research, a hexahedral mesh was used to divide the rail and subdivide the cracks. The local mesh diagram of the steel rail model in Figure 3 [15], and the ANSYS rail model is shown in Figure 4 [17,18].

## 3. Influence of Different Factors on Rail Fatigue Contact

### 3.1. Effect of Crack Length on Rail Fatigue Contact

**Figure 5:**
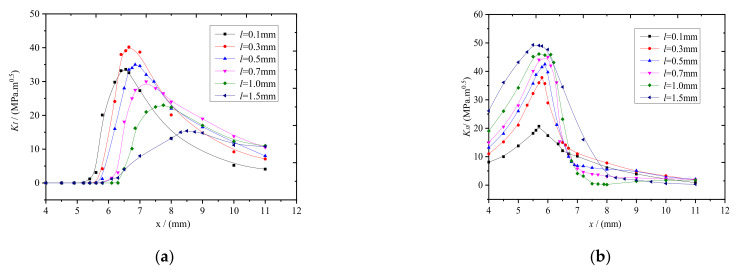
Positional relationships between and contact spot. (**a**) $K_{I}$; (**b**) $K_{II}$.

The constant axle load was set to 15 t, and the crack lengths were 0.1, 0.3, 0.5, 0.7, 1.0, and 1.5 mm. The stress field intensity analysis clearly revealed mainly two modes of stress intensity factors, $K_{I}$ and $K_{II}$ (where $K_{II}$ is the stress intensity factor for a opening crack at the rail crack). The positional relationships between $K_{I}$, $K_{II}$ and contact spot are shown in Figure 5, where x represents the distance between the center of the contact spot and the crack.

As shown in Figure 5a, for a constant axle load of 15 t, the maximum of the mode I crack continuously moved toward the edge of the contact spot with increasing rail tread crack length (*l*). When *l* ≤ and > 0.5 mm, the maximum $K_{I}$ was concentrated at the inner and outer edges of the rail contact spot, respectively.

As shown in Figure 5b, for a constant axle load of 15 t, the maximum $K_{II}$ was only slightly displaced (remaining in the range 5.5−6.1 mm between the rail contact spot) with increasing l compared with $K_{I}$. Clearly, the maximum $K_{II}$ concentrated at the center of the rail contact spot, which is where mode II failure occurs, with increasing l.

**Figure 6:**
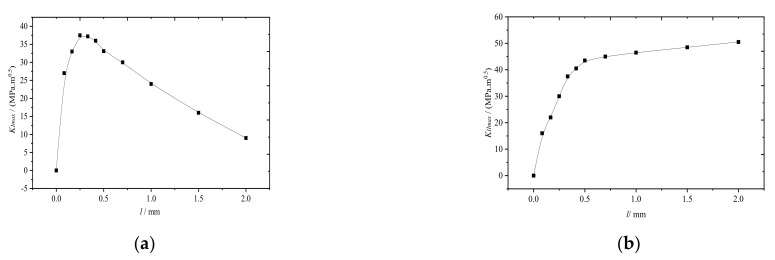
$K_{Imax}$ and $K_{IImax}$ plotted as functions of l. (**a**) $K_{Imax}$ plotted as functions of *l*; (**b**) $K_{IImax}$ plotted as functions of l.

To elucidate the influence of the change in l on the growth of rail surface fatigue cracks, the maximum stress intensity factors ($K_{Imax}$ and $K_{IImax}$) were extracted for different crack lengths. For a constant axle load of 15 t, $K_{I}$ and $K_{II}$ are plotted as functions of l, as shown in Figure 6.

As shown in Figure 6a, the maximum $K_{I}$ ($K_{Imax}$: the maximum opening mode crack stress intensity factor) first increased rapidly and then decreased slowly with increasing, reaching the maximum when l=0.3 mm owing to the distribution of the vertical compressive stress on the rail surface and the maximum tensile stress at the rail tread. Furthermore, the magnitude of the tensile stress decreased with increasing rail length. As the crack length continued to increase, the rail was compressed, which closed the crack. As shown in Figure 6b, the maximum $K_{II}$ ($K_{IImax}$: the maximum sliding mode crack stress intensity factor) first increased rapidly and then slowly with increasing owing to the influence of the shear stress distribution of the rail tread. The maximum shear stress generated by the rail contact usually occurred at the edge of the rail contact spot approximately 4 mm from the surface.

In summary, wheel-rail rolling contacts generate both opening mode and sliding cracks, which are mode I-II cracks.

The main factors controlling the crack mode also varied with the change of l. For example, for l less than 0.3 mm, $K_{Imax}$ increased faster than $K_{IImax}$, therefore, the crack propagation was dominated by the opening mode cracks. When l more than 0.3 mm, on the other hand, $K_{Imax}$ gradually decreased while $K_{IImax}$ continued to slowly increase, suggesting that $K_{IImax}$ was superior to $K_{Imax}$. Therefore, the crack propagation was dominated by the sliding mode cracks. As the crack length continued to increase, $K_{I}$ tended to 0, and the crack propagation mode converted from mode I−II cracks to mode II ones.

The propagation rate of rail crack can be expressed by, as given in Equation (1).

(1) $$\frac{da}{dN}=C{\left(\Delta K\right)}^{m}$$

where ΔK is the stress intensity factor range, and C and m are rail material−related constant coefficients.

**Figure 7:**
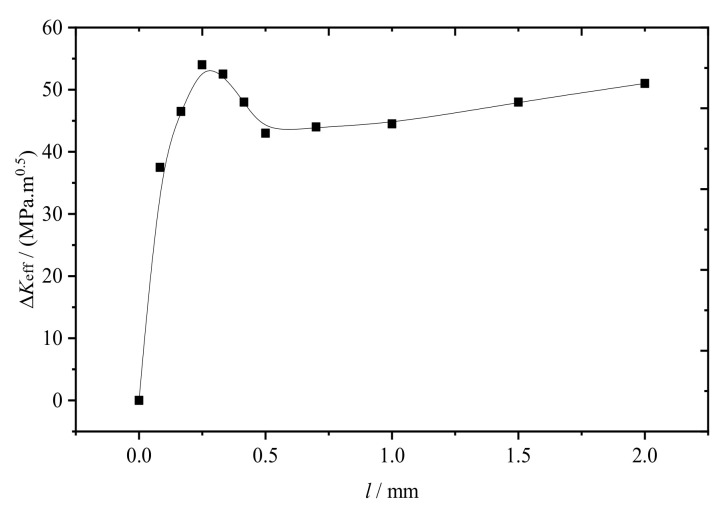
$\Delta K_{eff}$ plotted as a function of l.

For crack propagation induced by cyclic loading in I−II mixed mode, $\Delta K_{eff}$ can be used instead of ΔK. $\Delta K_{eff}$ is plotted as functions of l, as shown in Figure 7 [8,19].

Figure 7 clearly shows that as increased, $\Delta K_{eff}$ first increased rapidly, reaching the maximum when l≈0.3 mm, and then decreased. When the l=0.5 mm, $\Delta K_{eff}$ began slowly increasing. When l was in the range 0.3−0.5 mm, the crack propagated to the rail tread and damaged the rail by peeling off bits of rail material.

### 3.2. Effect of Rail Crack Propagation Angle on Wheel-Rail Contact Fatigue

**Figure 8:**
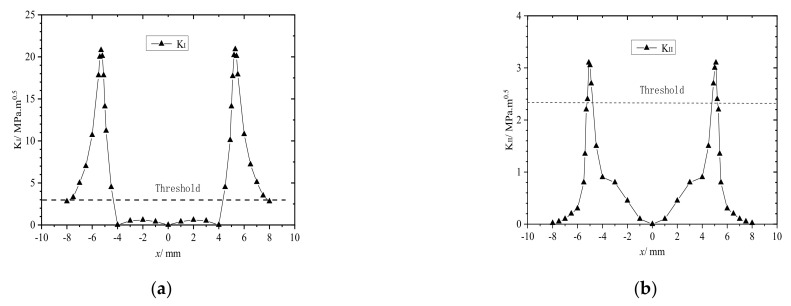
$K_{I}$ and $K_{II}$ plotted as functions of distance $\left(x\right)$ between the center point of rail−contact spot and rail tread crack (θ=90°) for steel train wheel passing over the crack. (**a**) $K_{I}$ plotted as functions of distance $\left(x\right)$ between the center point of rail contact spot and rail tread crack (θ=90°) for steel train wheel passing over crack; (**b**) $K_{II}$ plotted as functions of distance $\left(x\right)$ between the center point of rail contact spot and rail tread crack (θ=90°) for steel train wheel passing over the crack.

**Figure 9:**
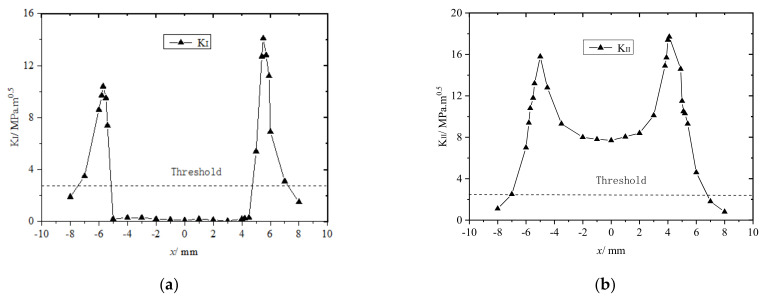
$K_{I}$ and $K_{II}$ plotted as functions of distance $\left(x\right)$ between the center point of rail−contact spot and rail tread crack (θ=45°) for steel train wheel passing over the crack. (**a**) $K_{I}$ plotted as functions of distance $\left(x\right)$ between the center point of rail−contact spot and rail tread crack (θ=45°) for steel train wheel passing over crack; (**b**) $K_{II}$ plotted as functions of distance $\left(x\right)$ between the center point of rail−contact spot and rail tread crack (θ=45°) for steel train wheel passing over the crack.

The axle load was set to 10 t, and the angle between the rail crack and the train−wheel running direction (θ) was set to either 90° or 45°. While the train wheel passed over the crack on the rail tread, $K_{I}$ and $K_{II}$ varied with the distance between the center point of the rail contact spot and the crack at θ=90° and 45°, as shown in Figure 8 and Figure 9, respectively.

As shown in Figure 8, as the train wheel ran over the rail tread crack, horizontal friction and normal squeezing opened or closed the crack. Clearly, when the train wheel approached or moved away from the rail surface crack, the crack opened under tension, when the train wheel pressed down on the surface crack, the crack closed because the wheel and rail squeezed one other. In addition, when θ=90°, $K_{I}$ and $K_{II}$ both varied symmetrically with the change in the distance between the rail contact point and the crack. As shown in Figure 8a, when the rail contact spot was approaching the crack between −8 and −4 mm, $K_{I}$ first increased and then rapidly decreased to approximately 0. When the contact spot was passing over the crack between −4 and +4 mm, $K_{I}\approx 0$, and the crack closed. When the rail contact spot was moving away from the crack between +4 and +8 mm, $K_{I}$ first increased and then decreased rapidly. Therefore, the maximum $K_{I}$ was at the edge of the rail contact spot. When the rail contact spot was directly over the rail crack, $K_{I}=0$, and the crack did not propagate. Although $K_{II}$ varied similarly to $K_{I}$, the change was less prominent, and $K_{II}=0$ only when the contact spot was directly pressing down on the crack.

When θ=45°, $K_{I}$ and $K_{II}$ both varied asymmetrically as the distance between the center point of the rail contact spot and the rail crack changed, as shown in Figure 9. In contrast to the crack at θ=90°, the maximum $K_{I}$ when the wheel−rail contact spot was moving away from the crack was higher than that when the spot was moving toward the crack. Although the overall trend was the same when the rail contact spot was at or near the rail cracks, $K_{I}\approx 0$, and the rail crack did not propagate. In contrast to the crack at θ=90°, the maximum $K_{II}$ was higher, and $K_{II}\neq 0$ and was well above the threshold required for the crack to propagate, so the crack always remained open. Figure 8b and Figure 9b clearly show that the maximum $K_{II}$ for the crack at θ=45° was higher than that for the one at θ=90°. When the rail contact spot was directly over the crack, $K_{II}$ for the crack at θ=90° was obviously below the threshold required for the crack to propagate because it did not, whereas $K_{II}$ for the crack at θ=45° was obviously above the threshold because the crack did propagate. The analysis clearly shows that when train wheels pass over both oblique (45°) and transverse (90°) rail tread cracks, the rail fatigue cracks propagate as mode I−II cracks, and the main crack propagation method is mode II when the rail contact spot falls on or near the rail crack.

**Figure 10:**
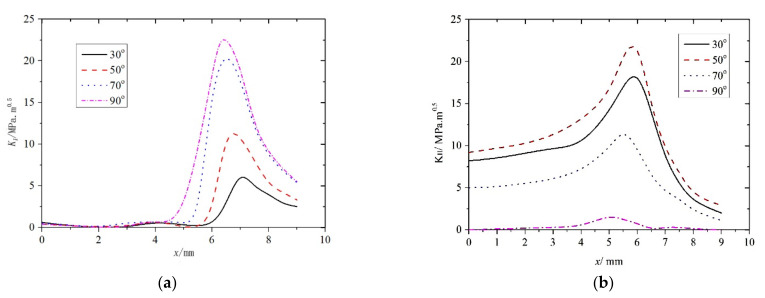
Stress intensity factors measured under frictionless conditions for different θ. (**a**) $K_{I}$; (**b**) $K_{II}$.

**Figure 11:**
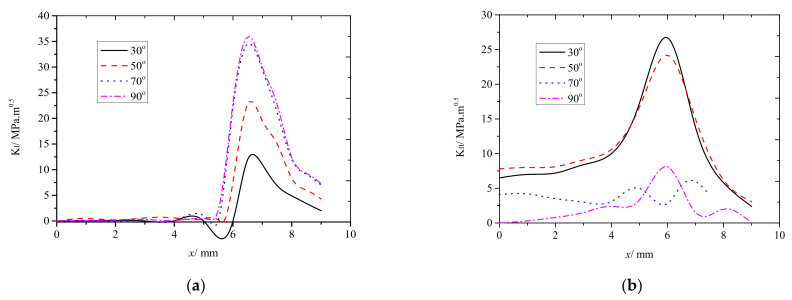
Stress intensity factors measured under friction rolling conditions for different θ. (**a**) $K_{I}$; (**b**) $K_{II}$.

Figure 8 and Figure 9 both show that the maximum K (where K is the stress intensity factor) when the rail contact spot was moving away from the crack was higher than that when the spot was moving toward the crack. Therefore, K was only analyzed when the wheel−rail contact spot was moving away from the crack, which reduced the number of computer calculations accordingly. The axle load was set to 15 t under both frictionless and rolling conditions and the changes in both $K_{I}$ and $K_{II}$ were studied while the distance between the center of the contact spot and the rail crack changed when θ=30°, 50°, 70°, and 90°. The results are presented in Figure 10 and Figure 11.

**Figure 12:**
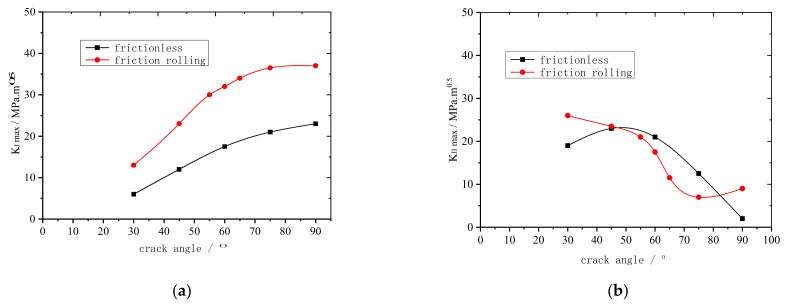
$K_{Imax}$ and $K_{IImax}$ plotted as functions of θ for different operating conditions. (**a**) $K_{Imax}$ plotted as functions of θ for different operating conditions; (**b**) $K_{II}max$ plotted as functions of θ for different operating conditions.

Figure 11a and Figure 12a clearly show that although $K_{I}$ varied differently under smooth (i.e., frictionless) and friction rolling conditions, the overall trend was identical. When the center of the rail contact was directly over the crack, $K_{I}\approx 0$ and the rail crack did not propagate regardless of the contact conditions and θ. Under frictionless conditions, the maximum $K_{I}$ was 6.6 mm away from the crack. Under friction rolling conditions, the maximum $K_{I}$ was 6.4 mm away from the crack, therefore, the maximum $K_{I}$ was at the edge of the contact spot under both conditions. Figure 10b and Figure 11b clearly show that for different contact conditions and θ, although $K_{II}$ varied slightly more chaotically than $K_{I}$, they both showed similar variation trends. The maximum $K_{II}$ was 6.0 mm and 6.2 mm away from the crack, therefore, the maximum $K_{II}$ was at the edge of the contact spot.

To further elucidate the effects of both the contact conditions and θ on the fatigue crack growth, the maximum $K_{I}$ and $K_{II}$ were analyzed in more detail. The changes in both $K_{Imax}$ and $K_{IImax}$ with increasing θ were measured under different operating conditions. The results are shown in Figure 12.

As shown in Figure 12, $K_{Imax}$ increased with increasing θ regardless of the operating conditions. Because horizontal friction can increase tensile stress at the edge of the contact spot, $K_{Imax}$ was significantly higher under friction rolling conditions than under frictionless ones. When θ between 30° and 70°, $K_{Imax}$ increased rapidly and for θ between 70° and 90°, $K_{Imax}$ increased slowly until reaching the maximum at θ=90°. $K_{IImax}$ varied slightly more chaotically than $K_{Imax}$, that is, $K_{IImax}$ decreased with increasing θ in the range 30−70°, where most of the main cracks had propagated.

To sum up, regardless of θ and the contact conditions, most of the cracks were mode I−II cracks, and the main crack modes were different. For example, When θ was less than 30°, the rail fatigue cracks were mainly mode II cracks. When θ was in the range 30−70°, the rail cracks were mode I−II cracks, and no major cracks propagated because $K_{I}$ and $K_{II}$ were almost identical. When θ was more than 70°, the fatigue cracks mainly propagated as mode I cracks.

Previously, the influence of θ on the propagation of rail fatigue cracks was studied, and the following analysis was conducted from the perspective of the propagation rate of rail crack. The analysis clearly shows that rail fatigue cracks are not single mode cracks but are mode I−II cracks. The stress intensity factor of mixed−mode cracks can be expressed by $K_{I+II}$, as given by Equation (2).

(2) $$K_{I+II}=K_{I}+K_{II}$$

**Figure 13:**
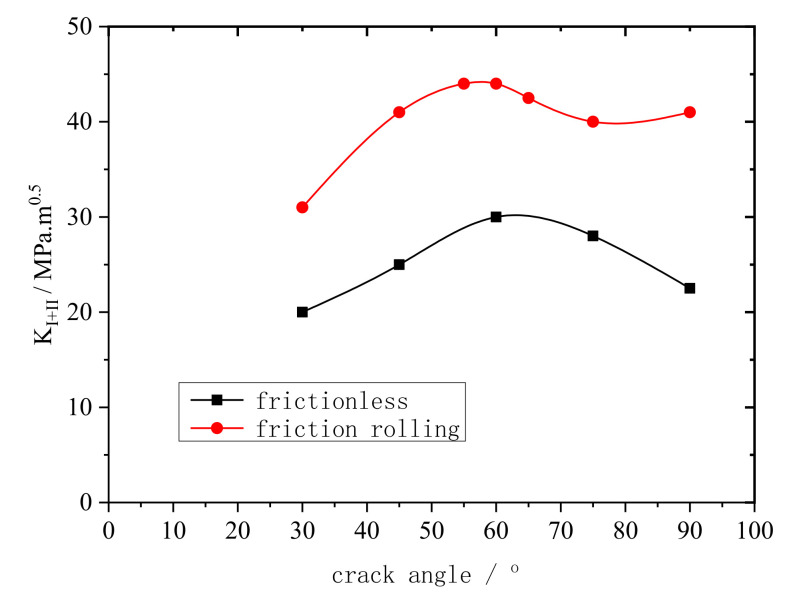
$K_{I+II}$ plotted as functions of θ for frictionless and friction rolling conditions.

$K_{I+II}$ was measured under a 15 t vertical load and is plotted as functions of θ for the frictionless and friction rolling conditions, as shown in Figure 13.

As shown in Figure 13, under both the frictionless and friction rolling conditions, $K_{I+II}{}_{}$ first increases and then decreases gradually. The overall trend is relatively stable, and the maximum $K_{I+II}$ is at θ=60°. Thus, when θ=60°, fatigue cracks propagate the fastest. These results suggest that fracture cracks propagate the easiest when θ=60° (i.e., θ=60° is the most dangerous rail crack propagation angle), which provides a basis for predicting crack propagation life.

## 4. Conclusions

In railway transportation, steel rails are prone to the formation of fatigue microcracks under the cyclic loads of train wheels, and such cracks further propagate with accumulating fatigue. Therefore, this paper analyzed the crack propagation of rail tread from microcracks to macrocracks, and investigated the influence of several main factors including rail crack length and crack propagation angle during fatigue crack propagation. The main findings are as follows:

1. When the rail crack length was less than 0.3 mm, mode I crack propagation was dominant. When the rail crack length was more than 0.3 mm, mode II crack propagation was dominant. When the rail crack length was between 0.3 and 0.5 mm, the rail tread material peeled and broke off.
2. Regardless of the crack propagation angle and contact conditions, the rail cracks were mainly mode I–II cracks. However, the main crack modes were different. When the crack propagation angle was less than 30°, mode II cracks were dominant. When the crack propagation angle was between 30 and 70°, the cracks were mainly mode I–II cracks. When the crack propagation angle was more than 70°, mode I cracks were dominant. Regardless of the motion conditions, the maximum equivalent stress intensity factor was at 60°. Therefore, when the crack propagation angle was 60°, the rail cracks propagated the fastest.

Due to time constraints, the results of this article are not particularly perfect, and in the future, the dynamic crack propagation of pre-existing cracks will be simulated by using the three-dimensional wheel–rail contact model.

## Figures and Tables

**Table 1:** Wheel–rail material performance parameters.

Material	Elastic Modulus MPa	Poisson’s Ratio	Yield Limit MPa	Tangential Modulus MPa	Threshold MPa.m^0.5^	Fracture Toughness MPa.m^0.5^
U71Mn	210,000	0.3	550	21,000	2.2	47
SMA490B	210,000	0.3	365	-	-	-

## Data Availability

Not applicable.
